# The Value of Routine Biopsy during Percutaneous Kyphoplasty for Vertebral Compression Fractures

**DOI:** 10.1371/journal.pone.0115417

**Published:** 2014-12-19

**Authors:** Qiang Li, Surong Hua, Chu Wang, Siyi Cai, Jia Zhang

**Affiliations:** 1 Department of Orthopedics, Peking Union Medical College Hospital, Beijing, China; 2 Department of Surgery, Peking Union Medical College Hospital, Beijing, China; University of California, San Diego, United States of America

## Abstract

**Objective:**

Percutaneous kyphoplasty (PKP) is now widely performed to treat VCF, which is usually caused by osteoporosis. Previous researches have reported unsuspected malignancies found by biopsy. However, the safety and cost-effective profiles of routine biopsy during PKP are unclear. The purpose of this study was to evaluate the feasibility of routine biopsy during PKP in treatment of VCF.

**Methods:**

Ninety-three patients (September 2007–November 2010) undergoing PKP without biopsy were reviewed as the control group. One hundred and three consecutive patients (November 2010–September 2013) undergoing PKP with biopsy of every operated vertebral level were prospectively enrolled as the biopsy group. The rate of unsuspected lesions was reported, and the severe adverse events, surgical duration, cement leakage rate and pain control were compared between the two groups.

**Results:**

No statistically significant differences were found between the two groups, regarding the severe adverse events, surgical duration, cement leakage rate and pain control. Four unsuspected lesions were found in the biopsy group, three of which were malignancies with a 2.9% (3/103) unsuspected malignancy rate. The economic analysis showed that routine biopsy was cost-effective in finding new malignancies comparing with a routine cancer screening campaign.

**Conclusions:**

Routine biopsy during PKP was safe and cost-effective in finding unsuspected malignancies. We advocate routine biopsy in every operated vertebral level during PKP for VCF patients.

## Introduction

Vertebral compression fractures (VCF) are the most common osteoporotic fractures, and one of the most common injuries all over the world with an aging population [1]. In the United States, there were about 700,000 patients diagnosed with VCF annually, and the prevalence was as high as 18% and 22% in men and women over the age of 55 years respectively [2]. The European Prospective Osteoporosis Study (EPOS) showed that the incidence of VCF in European men and women aged 50–79 years was 5.7/1000 and 9.9/1000 per year respectively [3]. VCF can cause pain, deformity and other clinical symptoms. About 20–30% of the VCF cases would still have chronic pain after conservative treatment, and require operation eventually [4]. Minimally invasive techniques such as percutaneous kyphoplasty (PKP) and percutaneous vertebroplasty (PVP) were increasingly used in the treatment for chronic pain and disabilities caused by VCF in recent years, and proven as effective and safe methods [5]. PKP was found to be cost effective, and perhaps even cost saving, compared with PVP or nonsurgical management among VCF patients [6], [7], [8], [9]. Significant pain relief and life quality improvement after PKP were reported in literature [10], [11].

Most VCF are caused by osteoporosis, while other metabolic diseases or malignancies may also cause pathological fractures. The underlying etiology of vertebral collapse may influence the decision to treat and the method of treatment. The preoperative diagnosis of osteoporotic VCF is made based on clinical manifestations and radiologic findings; however, this does not always reveal the true etiology of a VCF. Previous researches have described unsuspected malignancies found in bone biopsies during PKP or PVP [12], [13], [14], but routine biopsy was not use in some of these researches; what's more, none of them were case-control studies.

The aim of this study was to evaluate the feasibility of routine biopsy during PKP by comparing the safety and cost-effective data between the biopsy group and the control group.

## Methods

### Study design

All patients were treated by one surgeon from the Department of Orthopedics, Peking Union Medical College Hospital, Beijing, China. The retrospectively maintained database from September 2007 to November 2010 was interrogated to determine the baseline incidence of adverse events in VCF patients undergoing PKP without biopsy (control group). Following the introduction of routine biopsy in November 2010, an investigation into the clinical and radiologic outcomes in a prospective cohort was performed (biopsy group, November 2010–September 2013). All patients were evaluated with magnetic resonance imaging (MRI) preoperatively, and anteroposterior and lateral radiographs before and after the operation. Indications for PKP were: (1) focal back pain related to the location of the VCF on MRI, and (2) visual analogue scale (VAS) of pain over 6 of 10 after at least 6 weeks of conservative treatment. Exclusion criteria were coagulation disorders, bone cement allergies and local infection. Unsuspected lesions found by biopsy were recorded, and the surgical duration (reported as operative time per vertebra), cement leakage rate, pain control (recorded as VAS before and 3 days after the operation; change of VAS was calculated as preoperative VAS minus postoperative VAS) and cost-effective data were compared between the two groups.

The study protocol was reviewed and approved by the Ethics Committee of Peking Union Medical College Hospital. Written informed consent was obtained from all participants.

### Control group

One hundred and thirty-nine vertebrae from 93 non-consecutive patients undergoing PKP without biopsy were reviewed as the control group. Patients with selective biopsy for suspected malignancy or other reasons during the same period were excluded. Of these 93 patients, 66 were females (71.0%). The mean age was 68.0±9.7 years (range 50 to 87). There were 55 patients undergoing one-level PKP, 30 patients two-level, and 8 patients three-level. The 139 vertebral levels included: T5 (1), T6 (3), T7 (5), T8 (3), T9 (8), T10 (7), T11 (12), T12 (26), L1 (36), L2 (16), L3 (15), L4 (5) and L5 (2).

### Biopsy group

One hundred and fifty-seven vertebrae from 103 consecutive patients undergoing PKP with biopsy of every operated vertebral level were prospectively enrolled as the biopsy group. Of these 103 patients, 72 were females (69.9%). The mean age was 68.7±9.9 years (range 50 to 89). There were 59 patients undergoing one-level PKP, 34 two-level, and 10 three-level. The 157 vertebral levels included: T4 (1), T5 (1), T6 (3), T7 (7), T8 (5), T9 (8), T10 (6), T11 (15), T12 (28), L1 (39), L2 (19), L3 (13), L4 (10) and L5 (2).

### Procedure

The standard PKP was performed under local anesthesia guided by a G-arm fluoroscopy with the patient placed in prone hyperextended position. A working channel was usually set through the transpedicular approach. An appropriate spinal needle (usually 14G/2.1 mm) was carefully advanced into the fractured vertebra, and the bone specimen was obtained using the trephine in the biopsy group. Histological evaluation was performed by a senior pathologist. Pathological examination fee and the trephine's price were recorded as the extra cost for the biopsy, which is about 118.1 U.S. dollars per vertebra (16.3 and 101.8 dollars respectively).

### Statistical analysis

Comparison between the two groups was carried out using chi-square analysis for categorical variables and two-tailed *t* test for continuous variables at the 5% level of significance. All analysis was done with SPSS 19.0 software (SPSS Inc, Chicago).

## Results

### Surgical outcomes

Technical success was achieved in all patients of both groups. No severe adverse events (defined as death or paraplegia) were reported. Demographic data, surgical duration, cement leakage rate and pain control were compared between the two groups (Table 1). The patient demographics were similar, as well as the operative time per vertebra and pain control, with no statistically significant differences found. We found a higher cement leakage rate in the biopsy group comparing with the control group, but it didn't reach a statistical significance (7.64% versus 6.47%, *P* = 0.675).

**Table 1:** Comparison of demographics, surgical duration, cement leakage rate and pain control between the biopsy and control groups.

	Control group	Biopsy group	*P* value
Age (years)	68.0±9.7	68.7±9.9	0.609
Female	71.0% (66/93)	69.9% (72/103)	0.871
Operative time per vertebrae (min)	40.1±7.0	41.1±6.6	0.245
Cement leakage rate	6.47% (9/139)	7.64% (12/157)	0.675
Preoperative VAS	7.41±0.80	7.36±0.86	0.679
Postoperative VAS	2.38±0.87	2.40±0.87	0.862
Change of VAS	5.03±0.99	4.96±1.04	0.626

Continuous data are presented as mean ± standard deviation; categorical data are given as percentage (counts).

VAS, visual analogue scale of pain.

### Unsuspected lesions

One hundred and fifty-one biopsies obtained from 151 vertebrae of 97 patients were suitable for histologic evaluation. Six biopsies (3.8%, 6/157) from 6 vertebra of 6 patients consisted of too little or crushed material, which could not be interpreted by the pathologist. In the group of 3 patients with a history of cancer (2 with lung cancer and 1 with kidney cancer) and no radiological suspicion of malignancy causing the VCF, no signs of malignant diseases were found in the biopsies. Meanwhile, there were 4 (2.5%, 4/157) biopsies showing unsuspected lesions, including 2 cases of multiple myeloma, 1 case of Paget's disease and 1 case of chronic osteomyelitis. All the 4 cases didn't have any related clinical manifestations or imaging suspicion, and were considered as the osteoporotic VCF preoperatively.

**Figure 1:**
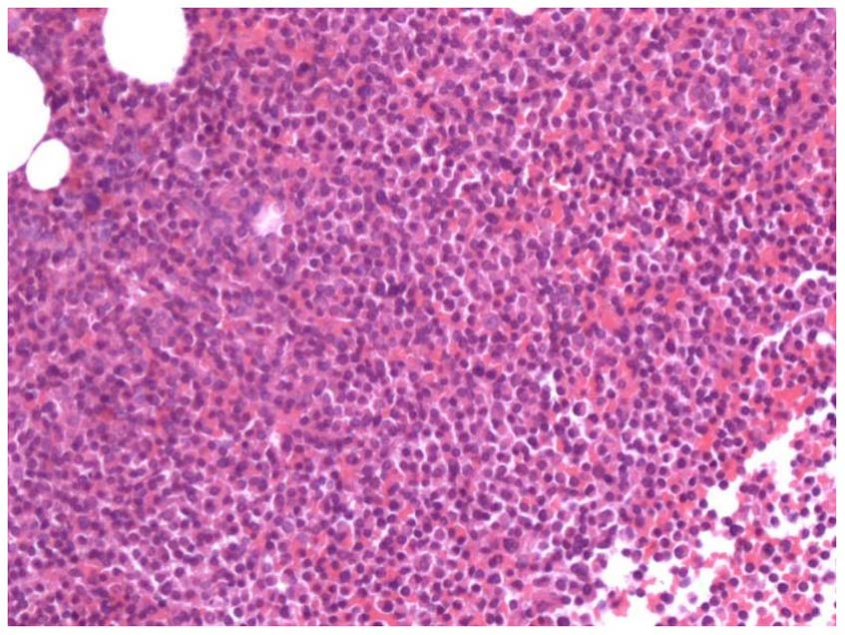
Vertebral bone biopsy from a patient with no history of malignancy showed clusters of plasma cells consistent with multiple myeloma. The immunohistochemical results were CD138 (+), CD20 (+), CD3 (−), CD38 (+), PC (+), with a Ki-67 index of 40% (hematoxylin and eosin, original magnification ×100).

Among the 4 unsuspected cases, three underwent one-level PKP, while the remaining one underwent three-level PKP. The first case of multiple myeloma (47-year-old female) was transferred to a hematologist. Three biopsies were obtained during the three-level PKP (T12–L2) from the second case of multiple myeloma (63-year-old female). Sign of multiple myeloma was only found in the T12 biopsy (Fig. 1), while the other two biopsies just revealed osteoporotic changes. The patient was suggested to a hematologist, but she did not show up in the hematology clinic. Two months later, this patient had a pathologic fracture on her right femoral shaft. We operated on the femur and the pathologic result showed multiple myeloma again. The case with Paget's disease of a 56-year-old female was suggested to medical therapy. Close observation was given to the patient with chronic osteomyelitis (73-year-old female), and no symptom of infection was found in the 6 month follow-up.

No other malignancies were found during the limited follow-up (mostly just 3–6 month) in both the control and biopsy groups.

### Cost-effective data

Unsuspected lesions were found in 2.5% (4/157) of the total biopsies. Considering the extra cost of 118.1 dollars per biopsy, the cost per unsuspected lesion found was about 4,724 dollars. Unsuspected malignancies were found in 1.9% (3/157) of the total biopsies, and the cost per unsuspected malignancy found was about 6,216 dollars. This cost was lower than the cost per breast cancer found (about 10,000 dollars; calculated as 20 dollars per person screened divided by 0.2% as the percentage of breast cancer found) in a routine screening campaign in China [15].

## Discussion

Given the aging of population and the prevalence of osteoporosis, VCF has become one of the most common injuries all over the world [16], [17]. Most VCF patients are presumed to have osteoporosis as the etiology. However, multiple myeloma, lymphoma and metastatic cancers are also prevalent in the same age-group [18], and osteoporotic VCF cannot be reliably diagnosed only based on clinical manifestations and radiologic findings. Misdiagnosis can lead to disastrous consequence. Previous researches have reported inconsistent unsuspected malignancy percentages found by biopsy during PKP or PVP, varying from 0.4% to 7.1% [12], [13], [14], [19], [20]. Some researchers recommended that a biopsy should be obtained in every vertebral augmentation procedure [12], [19], [20], while others suggested that the biopsy should only be performed in the first time augmentation [13]. It should be noticed that routine biopsy (in each patient or in each vertebra) was not use in some of these studies, and none of them were case-control studies to evaluate the safety and cost-effective profiles of the biopsy.

In the present case-control study, following the introduction of routine biopsy in every operated vertebral level, the severe adverse events, operative time per vertebra and the pain control results remained almost the same. The cement leakage rate was slightly higher in the biopsy group, but it didn't reach a statistical significance. These results confirmed the idea that biopsy was safe. Four unsuspected lesions were found in 157 vertebrae from 103 patients, three of which were malignancies. The 2.9% (3/103, calculated as malignancies divided by the total number of patients) unsuspected malignancy rate in the current study was higher than the 1.3% (3/238) unsuspected malignancy rate of lymphoma Shindle *et al* reported [20], the 0.7% (1/142) incidence of multiple myeloma Togawa *et al* reported [13], and the 0.4% (2/502) unsuspected malignancy rate reported by Zhang *et al* [14], but lower than the 7.1% (3/42) incidence of previously unsuspected malignancy reported by Schoenfeld *et al* [19], and the 3.8% (3/78) unsuspected malignancy rate reported by Muijs *et al* [12]. The differences in percentages of unsuspected malignancy may be explained by the size of the cohorts or differences in the criteria of biopsy. It is reasonable to presume that the malignancy rate should be higher in the selective biopsy cohorts than the routine biopsy ones.

Unlike the routine biopsy of one vertebral level in each patient used in most previous studies, routine biopsy in every operated vertebral level was used in the biopsy group in the current study. We found that osteoporosis and malignancy could present in different levels of vertebra in the same patient. This case told us that sometimes the biopsy of just one vertebral level in a multi-level VCF patient might not be enough. The economic analysis in this study showed that routine biopsy was cost-effective, and perhaps even cost-saving in finding new malignancies comparing with a routine cancer screening campaign in China. A traditional economic comparison in the cost per life-year gained or per quality-adjusted life year (QALY) gained was not applicative here for the reason that the malignancies found in the biopsies were heterogeneous. Considering the safety and the low cost of biopsy during PKP, we thought that the possible advantages of early detection of malignant disease outweighed the risks. Therefore we advise that biopsies should be carried out in every VCF level for which PKP is indicated.

Several points should be noticed in this present study. First, it was not randomized, and the biopsy group was compared to a historical control group. Therefore, any observations regarding surgical duration and cement leakage rates could not rule out the confounding factor of increased experience. Second, this cohort was relatively young (mean age <70 years) and mainly consisted of women (about 70%), and all three unsuspected malignancies were also found in relatively young females (47, 56 and 63 years old, respectively). VCF is a well-recognized consequence of postmenopausal bone loss in elderly women. We thought that the poor present conditions in osteoporosis prevention and control in Chinese women may contribute to the composition of this cohort, but there was no literature describing this phenomenon. Thus, the composition of VCF patients may be different in other countries.

## Conclusions

Our data suggested that routine biopsy during PKP was safe and cost-effective in finding unsuspected malignancies. We advocate routine biopsy in every operated vertebral level during PKP for VCF patients.

## Supporting Information

S1 Excel **The original data of the control group (non-biopsy group) in English.**
(XLSX)

S2 Excel **The original data of the biopsy group in English.**
(XLSX)

S3 Excel **The original data of the control group (non-biopsy group) in Chinese.**
(XLSX)

S4 Excel **The original data of the biopsy group in Chinese.**
(XLSX)

S1 SPSS **The original working dataset with the SPSS software (sorted by patient) in English.**
(SAV)

S2 SPSS **The original working dataset with the SPSS software (sorted by vertebra) in English.**
(SAV)

S3 SPSS **The original working dataset with the SPSS software (sorted by patient) in Chinese.**
(SAV)

S4 SPSS **The original working dataset with the SPSS software (sorted by vertebra) in Chinese.**
(SAV)

## References

[1] Ross PD (1997) Clinical consequences of vertebral fractures. Am J Med 103:30S–43S – 9302895 10.1016/s0002-9343(97)90025-5

[2] Riggs BL, Melton LR (1995) The worldwide problem of osteoporosis: insights afforded by epidemiology. Bone 17:505S–511S. 8573428 10.1016/8756-3282(95)00258-4

[3] Felsenberg D, Silman AJ, Lunt M, Armbrecht G, Ismail AA, et al (2002) Incidence of vertebral fracture in europe: results from the European Prospective Osteoporosis Study (EPOS). J Bone Miner Res 17:716–724. 11918229 10.1359/jbmr.2002.17.4.716

[4] Cooper C, Atkinson EJ, O'Fallon WM, Melton LR (1992) Incidence of clinically diagnosed vertebral fractures: a population-based study in Rochester, Minnesota, 1985-1989. J Bone Miner Res 7:221–227. 1570766 10.1002/jbmr.5650070214

[5] Wardlaw D, Cummings SR, Van Meirhaeghe J, Bastian L, Tillman JB, et al (2009) Efficacy and safety of balloon kyphoplasty compared with non-surgical care for vertebral compression fracture (FREE): a randomised controlled trial. Lancet 373:1016–1024. 19246088 10.1016/S0140-6736(09)60010-6

[6] Edidin AA, Ong KL, Lau E, Schmier JK, Kemner JE, et al (2012) Cost-effectiveness analysis of treatments for vertebral compression fractures. Appl Health Econ Health Policy 10:273–284. 22591065 10.2165/11633220-000000000-00000

[7] Svedbom A, Alvares L, Cooper C, Marsh D, Strom O (2013) Balloon kyphoplasty compared to vertebroplasty and nonsurgical management in patients hospitalised with acute osteoporotic vertebral compression fracture: a UK cost-effectiveness analysis. Osteoporos Int 24:355–367. 22890362 10.1007/s00198-012-2102-y PMC3691631

[8] Berenson J, Pflugmacher R, Jarzem P, Zonder J, Schechtman K, et al (2011) Balloon kyphoplasty versus non-surgical fracture management for treatment of painful vertebral body compression fractures in patients with cancer: a multicentre, randomised controlled trial. Lancet Oncol 12:225–235. 21333599 10.1016/S1470-2045(11)70008-0

[9] Ma XL, Xing D, Ma JX, Xu WG, Wang J, et al (2012) Balloon kyphoplasty versus percutaneous vertebroplasty in treating osteoporotic vertebral compression fracture: grading the evidence through a systematic review and meta-analysis. Eur Spine J 21:1844–1859. 22832872 10.1007/s00586-012-2441-6 PMC3459117

[10] Ledlie JT, Renfro M (2003) Balloon kyphoplasty: one-year outcomes in vertebral body height restoration, chronic pain, and activity levels. J Neurosurg 98:36–42. 12546386 10.3171/spi.2003.98.1.0036

[11] Phillips FM, Ho E, Campbell-Hupp M, McNally T, Todd WF, et al (2003) Early radiographic and clinical results of balloon kyphoplasty for the treatment of osteoporotic vertebral compression fractures. Spine (Phila Pa 1976) 28:2260–2267 – 14520041 10.1097/01.BRS.0000085092.84097.7B

[12] Muijs SP, Akkermans PA, van Erkel AR, Dijkstra SD (2009) The value of routinely performing a bone biopsy during percutaneous vertebroplasty in treatment of osteoporotic vertebral compression fractures. Spine (Phila Pa 1976) 34:2395–2399. 19829253 10.1097/BRS.0b013e3181b8707e

[13] Togawa D, Lieberman IH, Bauer TW, Reinhardt MK, Kayanja MM (2005) Histological evaluation of biopsies obtained from vertebral compression fractures: unsuspected myeloma and osteomalacia. Spine (Phila Pa 1976) 30:781–786. 15803081 10.1097/01.brs.0000157478.03349.57

[14] Zhang L, Li J, Yang H, Luo Z, Zou J (2013) Histological evaluation of bone biopsy results during PVP or PKP of vertebral compression fractures. Oncol Lett 5:135–138. 23255908 10.3892/ol.2012.944 PMC3525491

[15] Li ZF, Wang SM, Shi JF, Zhao FH, Ma JF, et al (2012) Combined screening of cervical cancer, breast cancer and reproductive tract infections in rural China. Asian Pac J Cancer Prev 13:3529–3533. 22994789 10.7314/apjcp.2012.13.7.3529

[16] Leslie WD, Morin SN (2014) Osteoporosis epidemiology 2013: implications for diagnosis, risk assessment, and treatment. Curr Opin Rheumatol 26:440–446. 24807402 10.1097/BOR.0000000000000064

[17] Hernlund E, Svedbom A, Ivergard M, Compston J, Cooper C, et al (2013) Osteoporosis in the European Union: medical management, epidemiology and economic burden. A report prepared in collaboration with the International Osteoporosis Foundation (IOF) and the European Federation of Pharmaceutical Industry Associations (EFPIA). Arch Osteoporos 8:136. 24113837 10.1007/s11657-013-0136-1 PMC3880487

[18] Phekoo KJ, Schey SA, Richards MA, Bevan DH, Bell S, et al (2004) A population study to define the incidence and survival of multiple myeloma in a National Health Service Region in UK. Br J Haematol 127:299–304. 15491289 10.1111/j.1365-2141.2004.05207.x

[19] Schoenfeld AJ, Dinicola NJ, Ehrler DM, Koerber A, Paxos M, et al (2008) Retrospective review of biopsy results following percutaneous fixation of vertebral compression fractures. Injury 39:327–333. 17880977 10.1016/j.injury.2007.06.019

[20] Shindle MK, Tyler W, Edobor-Osula F, Gardner MJ, Shindle L, et al (2006) Unsuspected lymphoma diagnosed with use of biopsy during kyphoplasty. J Bone Joint Surg Am 88:2721–2724. 17142423 10.2106/JBJS.F.00100

